# Design and analysis of combined discrete-time zeroing neural network for solving time-varying nonlinear equation with robot application

**DOI:** 10.3389/fnbot.2025.1576473

**Published:** 2025-07-11

**Authors:** Zhisheng Ma, Shaobin Huang

**Affiliations:** College of Computer Science and Technology, Harbin Engineering University, Harbin, Heilongjiang, China

**Keywords:** discrete-time zeroing neural network, time-varying nonlinear equation, Taylor difference formula, matrix inversion, robot manipulators

## Abstract

Zeroing neural network (ZNN) is viewed as an effective solution to time-varying nonlinear equation (TVNE). In this paper, a further study is shown by proposing a novel combined discrete-time ZNN (CDTZNN) model for solving TVNE. Specifically, a new difference formula, which is called the Taylor difference formula, is constructed for first-order derivative approximation by following Taylor series expansion. The Taylor difference formula is then used to discretize the continuous-time ZNN model in the previous study. The corresponding DTZNN model is obtained, where the direct Jacobian matrix inversion is required (being time consuming). Another DTZNN model for computing the inverse of Jacobian matrix is established to solve the aforementioned limitation. The novel CDTZNN model for solving the TVNE is thus developed by combining the two models. Theoretical analysis and numerical results demonstrate the efficacy of the proposed CDTZNN model. The CDTZNN applicability is further indicated by applying the proposed model to the motion planning of robot manipulators.

## 1 Introduction

Nonlinear equation (NE) has an important implication in many fields of science and engineering, such as signal and image processing, pattern recognition, and robot motion planning. Many solutions for the NE have been reported (Liao et al., 2020; Wu et al., 2021; Zeng et al., 2024; Mathews and Fink, 2004; Ramos and Monteiro, 2017; Sharma et al., 2017; Zhang and Yi, 2011; Xiao and Lu, 2015; Zhang et al., 2017). The Newton method, which possesses the quadratic convergence, was the classic iteration method to find the solution of the NE (Mathews and Fink, 2004). On the basis of Newton method, a new approach was developed by Ramos and Monteiro (2017) to solve the NE. A class of multipoint methods for solving the NE was presented by Sharma et al. (2017). Different from these iteration methods, the methods based on recurrent neural network (RNN) were designed to solve the NE. The gradient neural network (GNN), which possesses the exponential convergence, was the classic RNN model for determining the roots of the NE (Zhang and Yi, 2011). As shown by Xiao and Lu (2015), the GNN was accelerated to finite convergence through a special activation function. Another typical RNN model termed the zeroing neural network (ZNN) was provided by Zhang et al. (2017), and it was indicated to have a connection to Davidenko method in solving the NE. Notably, the abovementioned studies were reported on computing the static NE.

Focusing on solving the time-varying NE (TVNE), a continuous-time ZNN (CTZNN) model was developed by Zhang et al. (2012a), of which the effectiveness was substantiated by theoretical analysis and simulations. The nonlinearly-activated CTZNN model with finite-time convergence was designed by Xiao (2016) to solve the TVNE. Considering the existence of noise in the process of solving the TVNE, Jin et al. (2017b) presented an integration-enhanced CTZNN model, Li et al. (2020) provided a finite-time convergent and noise-rejection CTZNN model, and Dai et al. (2024) developed a norm-based CTZNN model with strong robustness. For possible digital hardware implementation, the discrete-time ZNN (DTZNN) model was investigated by Zhang et al. (2015) using the Euler difference formula to discretize the original CTZNN model. The DTZNN model was theoretically analyzed to have a square characteristic. That is, the steady-state computational error (SSCE) was expressed in the form of *O*(σ^2^), where σ denotes the sampling gap. Furthermore, the Broyden method was utilized to avoid the matrix inversion involved in the DTZNN model, and the modified DTZNN model was presented by Zhang et al. (2012b). This model was immune to singularity and still had the square characteristic.

Being different from the Euler difference formula, various Taylor-type difference formulas have been constructed (Liao et al., 2016; Guo et al., 2018; Xiang et al., 2019; Tang and Zhang, 2023; Cang et al., 2024; Chen et al., 2024; Xiang et al., 2025; Guo et al., 2025). For example, the Taylor difference formula with the *O*(σ^2^) truncation error was presented in Liao et al. (2016), while the one with *O*(σ^3^) truncation error was provided in Tang and Zhang (2023). These difference formula have been proven to be effective on the ZNN disretization. Specific for the TVNE solving, the Taylor difference formula in Guo et al. (2018) was used to discretize the CTZNN model (Zhang et al., 2012a). The resultant DTZNN model was developed, of which the SSCE is expressed in the form of *O*(σ^4^) (Guo et al., 2018). In Cang et al. (2024), the DTZNN model with the *O*(σ^5^) SSCE mode was designed to solve the TVNE. Notably, the shortcoming of such two DTZNN models is the requirement of calculating the inverse of Jacobian matrix, which could be (very) time consuming. In this sense, the DTZNN models in Guo et al. (2018) and Cang et al. (2024) may be less effective for the online solution of TVNE. Thus, an effective DTZNN model with great computational performance but without requirement of direct Jacobian matrix inversion should be designed and studied.

In this paper, motivated by the inspiring studies (Zhang et al., 2015, 2012b; Guo et al., 2018; Cang et al., 2024), a further investigation is provided by proposing a novel combined DTZNN (CDTZNN) model to solve the TVNE. Specifically, for the possible discretization of a continuous-time model, a new difference formula, which is called the Taylor difference formula, is developed by following Taylor series expansion (Mathews and Fink, 2004). Using the new formula to discretize the original CTZNN model (Zhang et al., 2012a) yields the resultant DTZNN model. On the basis of Guo et al. (2017), another DTZNN model is established for the Jacobian matrix inversion. With the combination of two models, the novel CDTZNN model for solving the TVNE is thus developed. Such a model does not require calculating the inverse of Jacobian matrix and can thus reduce the amount of calculation. Theoretical analysis and numerical results substantiate the efficacy of the proposed CDTZNN model.

In recent years, robot manipulators have attracted considerable attention in engineering applications (Li et al., 2020; Guo et al., 2018; Cang et al., 2024; Guo et al., 2017; Jin et al., 2017a, 2024; Yan et al., 2024; Zhang et al., 2025; Li et al., 2019; Xiao et al., 2019). The motion planning of robot manipulators, as a fundamental issue, has been widely investigated. Mathematically, the purpose of motion planning can be achieved when the corresponding TVNE is solved (Li et al., 2020; Guo et al., 2018; Cang et al., 2024). In consideration of this point, the proposed CDTZNN model is further studied to the motion planning of robot manipulators through solving the TVNE. On the basis of a DOBOT manipulator (Xiao et al., 2019), the simulation results are provided to validate the applicability of the proposed CDTZNN model.

The rest of the paper consists of five parts. Section 2 presents the previous studies on TVNE solving. Section 3 shows the Taylor difference formula. Section 4 describes the CDTZNN model and analyzes its computational performance. Section 5 provides the numerical results of the proposed CDTZNN model and applies such a model to robot manipulators. Section 6 concludes the study. The main contributions are listed as below.

1) A new Taylor difference formula is developed via the Taylor series expansion. Such a formula differs from the formulas used in Zhang et al. (2015, 2012b); Guo et al. (2018); Cang et al. (2024) for the original CTZNN discretization on solving the TVNE.
2) On the basis of the new Taylor difference formula, a novel CDTZNN model is proposed and studied to solve the TVNE. To the best of the authors' knowledge, such a CDTZNN model has not been reported yet.
3) The computational performance of the proposed CDTZNN model is analyzed theoretically. The CDTZNN efficacy is further substantiated by numerical results. The results also express the *O*(σ^4^) mode in the SSCE of the proposed model.
4) The proposed CDTZNN model is effectively applied to the motion planning of robot manipulators. The simulations under the DOBOT manipulator show the potential application of CDTZNN.

## 2 Previous studies on TVNE solving

This section shows the problem formulation of solving the TVNE. Then, the CTZNN and DTZNN models in the previous studies are presented.

### 2.1 Problem statement

In this study, the following TVNE is considered (Zhang et al., 2012a):


(1) $$\begin{array}{l} \text{f}\left({\text{x}}^{*}\left(t\right),t\right)=0\in {\mathbb{R}}^{n},\;\forall t\in \left[0,+\infty \right), \end{array}$$

where **f**(·):ℝ^*n*^ → ℝ^*n*^ denotes a differentiable nonlinear mapping and x^*^(*t*) ∈ ℝ^*n*^ denotes the theoretical solution of Equation 1. This study aims to find the numerical solution **x**(*t*) ∈ ℝ^*n*^ that holds the TVNE (Equation 1) true; specifically, as time *t* evolves, **x**(*t*) → x^*^(*t*).

### 2.2 CTZNN model

To effectively solve the TVNE (Equation 1), the following error function must converge to zero as time evolves:


(2) $$\begin{array}{l} \text{e}\left(t\right)=\text{f}\left(\text{x}\left(t\right),t\right)\in {\mathbb{R}}^{n}. \end{array}$$

In Zhang et al. (2012a), the decay formula $\dot{\text{e}}\left(t\right)=-\gamma \text{e}\left(t\right)$ is used, and the CTZNN model for solving the TVNE (Equation 1) is designed as follows:


(3) $$\begin{array}{l} \dot{\text{x}}\left(t\right)=-J^{-}\left(\text{x}\left(t\right),t\right)({\text{f}}_{t}\left(\text{x}\left(t\right),t\right)+\gamma \text{f}\left(\text{x}\left(t\right),t\right)), \end{array}$$

where $\dot{\text{e}}\left(t\right)$ and $\dot{\text{x}}\left(t\right)$ correspond to the time derivatives of **e**(*t*) and **x**(*t*), *J*^−^(**x**(*t*), *t*) ∈ ℝ^*n*×*n*^ denotes the inverse of Jacobian matrix *J*(**x**(*t*), *t*) = ∂**f**/∂**x**, ${\text{f}}_{t}\left(\text{x}\left(t\right),t\right)=\partial \text{f}/\partial t\in {\mathbb{R}}^{n}$ denotes the system time-derivative vector, and γ > 0 ∈ ℝ denotes the parameter for scaling the CTZNN convergence rate.

**Lemma 1 (Zhang et al.**, **2012a****)**. *When a solvable TVNE* (*Equation 1*) *is considered, the state vector* **x**(*t*) *of the CTZNN model (**Equation 3**), starting from an initial state* **x**(0) *close sufficiently to* x^*^(0)*, converges to the theoretical solution* x^*^(*t*) *of (**Equation 1**). That is*, **x**(*t*) → x^*^(*t*) *as* *t* *evolves*.

### 2.3 DTZNN models

To discretize the CTZNN model (Equation 3), the following Euler difference formula (Mathews and Fink, 2004) can be used:


(4) $$\begin{array}{l} {\dot{\text{x}}}_{k}=\dot{\text{x}}\left(t_{k}=k\sigma \right)=\frac{{\text{x}}_{k+1}-{\text{x}}_{k}}{\sigma }+\text{O}\left(\sigma \right), \end{array}$$

where **x**_*k*_ = **x**(*t*_*k*_ = *kσ*) with the subscript *k* ∈ *N* and sampling gap σ ∈ (0, 1), and **O**(σ) ∈ ℝ^*n*^ denotes the truncation error vector with every element as *O*(σ). By using Equation 4 to discretize Equation 3, the Euler DTZNN model for solving the TVNE (Equation 1) is formulated as follows (Zhang et al., 2015):


(5) $$\begin{array}{l} {\text{x}}_{k+1}={\text{x}}_{k}-J^{-}\left({\text{x}}_{k},t_{k}\right)(\sigma {\text{f}}_{t}\left({\text{x}}_{k},t_{k}\right)+h\text{f}\left({\text{x}}_{k},t_{k}\right)), \end{array}$$

where *h* = γσ ∈ ℝ denotes the step size with γ defined as in Equation 3.

To avoid the calculation for the inverse of Jacobian matrix, the Euler DTZNN model (Equation 5) is improved in Zhang et al. (2012b) by using Broyden method (Broyden, 1965). Specifically, $J^{-}\left({\text{x}}_{k},t_{k}\right)$ is calculated and approximated through a matrix $M_{k}=M\left(t_{k}=k\sigma \right)\in {\mathbb{R}}^{n\times n}$, where the recursion for updating *M*_*k*_ is given by


(6) $$\begin{array}{l} M_{k}=M_{k-1}+\frac{\left({\text{u}}_{k-1}-M_{k-1}{\text{v}}_{k-1}\right){\text{u}}_{k-1}^{\text{T}}M_{k-1}}{{\text{u}}_{k-1}^{\text{T}}M_{k-1}{\text{v}}_{k-1}}, \end{array}$$

with ${\text{u}}_{k-1}={\text{x}}_{k}-{\text{x}}_{k-1}\in {\mathbb{R}}^{n}$ and ${\text{v}}_{k-1}=\text{f}\left({\text{x}}_{k},t_{k}\right)-\text{f}\left({\text{x}}_{k-1},t_{k}\right)\in {\mathbb{R}}^{n}$. On the basis of Equation 6, replacing $J^{-}\left({\text{x}}_{k},t_{k}\right)$ by *M*_*k*_ in Equation 5 yields the modified DTZNN model as follows:


(7) $$\begin{array}{l} {\text{x}}_{k+1}={\text{x}}_{k}-M_{k}(\sigma {\text{f}}_{t}\left({\text{x}}_{k},t_{k}\right)+h\text{f}\left({\text{x}}_{k},t_{k}\right)). \end{array}$$

For further discussion, on the basis of the error function **e**(*t*) in Equation 2, the SSCE is defined as $\lim_{k\to \infty }∥{\text{e}}_{k}{∥}_{2}=\lim_{k\to \infty }∥\text{f}\left({\text{x}}_{k},t_{k}\right){∥}_{2}$, where ||·||_2_ returns the Euclidean norm of a vector. Then, the computational performances of the DTZNN models (Equations 5, 7), are shown by the following lemma.

**Lemma 2 (Zhang et al.**, **2015**, **2012b****)**. *When a solvable TVNE (**Equation 1**) is considered, the SSCE of each of the DTZNN models (**Equations 5*, *7**) is of* *O*(σ^2^).

Being different from the Euler difference formula, the following Taylor difference formulas are constructed (Tang and Zhang, 2023; Guo et al., 2017):


(8) $$\begin{array}{l} {\dot{\text{x}}}_{k}=\frac{8{\text{x}}_{k+1}+{\text{x}}_{k}-6{\text{x}}_{k-1}-5{\text{x}}_{k-2}+2{\text{x}}_{k-3}}{18\sigma }+\text{O}\left(\sigma^{3}\right), \end{array}$$

(9) $$\begin{array}{l} {\dot{\text{x}}}_{k}=\frac{24{\text{x}}_{k+1}-5{\text{x}}_{k}-12{\text{x}}_{k-1}-6{\text{x}}_{k-2}-4{\text{x}}_{k-3}+3{\text{x}}_{k-4}}{48\sigma }+\text{O}\left(\sigma^{3}\right), \end{array}$$

where sampling gap σ ∈ (0, 1), and **O**(σ^3^) ∈ ℝ^*n*^ denotes the truncation error vector with every element as *O*(σ^3^). In Cang et al. (2024), another Taylor difference formula is expressed as follows:


(10) $$\begin{array}{l} {\dot{\text{x}}}_{k}=\frac{110{\text{x}}_{k+1}+42{\text{x}}_{k}-89{\text{x}}_{k-1}-102{\text{x}}_{k-2}+12{\text{x}}_{k-3}+44{\text{x}}_{k-4}-17{\text{x}}_{k-5}}{276\sigma }+\text{O}\left(\sigma^{4}\right), \end{array}$$

where **O**(σ^4^) ∈ ℝ^*n*^ denotes the truncation error vector with every element as *O*(σ^4^). By using Equation 8 – 10 to discretize the CTZNN model (Equation 3), the following three Taylor DTZNN models for solving Equation 1 are obtained:


(11) $$\begin{array}{l} {\text{x}}_{k+1}=-\frac{1}{8}{\text{x}}_{k}+\frac{3}{4}{\text{x}}_{k-1}+\frac{5}{8}{\text{x}}_{k-2}-\frac{1}{4}{\text{x}}_{k-3}\\ \;-J^{-}\left({\text{x}}_{k},t_{k}\right)(\frac{9}{4}\sigma {\text{f}}_{t}\left({\text{x}}_{k},t_{k}\right)+h\text{f}\left({\text{x}}_{k},t_{k}\right)), \end{array}$$

(12) $$\begin{array}{l} {\text{x}}_{k+1}=\frac{5}{24}{\text{x}}_{k}+\frac{1}{2}{\text{x}}_{k-1}+\frac{1}{4}{\text{x}}_{k-2}+\frac{1}{6}{\text{x}}_{k-3}-\frac{1}{8}{\text{x}}_{k-4}\\ \;-J^{-}\left({\text{x}}_{k},t_{k}\right)(2\sigma {\text{f}}_{t}\left({\text{x}}_{k},t_{k}\right)+h\text{f}\left({\text{x}}_{k},t_{k}\right)), \end{array}$$

(13) $$\begin{array}{l} {\text{x}}_{k+1}=-\frac{21}{55}{\text{x}}_{k}+\frac{89}{110}{\text{x}}_{k-1}+\frac{51}{55}{\text{x}}_{k-2}-\frac{6}{55}{\text{x}}_{k-3}-\frac{2}{5}{\text{x}}_{k-4}\\ \;+\frac{17}{110}{\text{x}}_{k-5}-J^{-}\left({\text{x}}_{k},t_{k}\right)(\frac{138}{55}\sigma {\text{f}}_{t}\left({\text{x}}_{k},t_{k}\right)+h\text{f}\left({\text{x}}_{k},t_{k}\right)), \end{array}$$

where step size *h* are given by *h* = 9γσ/4 ∈ ℝ, *h* = 2γσ ∈ ℝ, and *h* = 138γσ/55 ∈ ℝ, respectively. Hereafter, Equation 11, 12, 13 are termed the Taylor DTZNN-I, DTZNN-II, and DTZNN-III models, respectively. Besides, the computational performance of such three models on solving the TVNE (Equation 1) is presented by the following lemma.

**Lemma 3 (Guo et al.**, **2018****; Cang et al.**, **2024****)**. *When a solvable TVNE (**Equation 1**) is considered, the SSCE of the Taylor DTZNN models (**Equations 11*, *12*, *13**) are of* *O*(σ^4^), *O*(σ^4^)*, and* *O*(σ^5^)*, respectively*.

According to Lemmas 2 and 3, the Taylor DTZNN models (Equations 11, 12, 13) can possess better computational performance than the DTZNN models (Equations 5, 7) in solving the TVNE (Equation 1) [in terms of *O*(σ^4^) and *O*(σ^5^) vs. *O*(σ^2^)]. However, the Taylor DTZNN models (Equation 11, 12, 13) requires calculating $J^{-}\left({\text{x}}_{k},t_{k}\right)$ at each iteration, which could be very time consuming. Thus, a novel DTZNN model that does not require the direct Jacobian matrix inversion is proposed in this work to solve the TVNE (Equation 1).

## 3 New Taylor difference formula

In this section, a new difference formula termed the Taylor difference formula is constructed to further investigate on discretizing a continuous-time model.

### 3.1 Scalar-valued form

The new Taylor difference formula depicted in a scalar-valued form is presented via the following theorem.

**Theorem 1**. *For effectively approximating the first-order derivative of the function* ϕ(·)*, the new Taylor difference formula is expressed as follows:*


(14) $$\begin{array}{l} \dot{\phi }\left(t_{k}\right)=\frac{36\phi \left(t_{k+1}\right)-\phi \left(t_{k}\right)-18\phi \left(t_{k-1}\right)-27\phi \left(t_{k-2}\right)+10\phi \left(t_{k-3}\right)}{78\sigma }\\ \;+O\left(\sigma^{3}\right), \end{array}$$

*with the subscript* *k* = 3, 4, 5, ⋯  *and sampling gap* σ ∈ (0, 1).

**Proof**. Assuming that ϕ ∈ *C*^4^[*a, b*] and that *t*_*k*+1_, *t*_*k*_, *t*_*k*−1_, *t*_*k*−2_, and *t*_*k*−3_ ∈ [*a, b*]. By following Taylor series expansion Mathews and Fink (2004), the expressions are obtained as follows:


(15) $$\begin{array}{l} \phi \left(t_{k+1}\right)=\phi \left(t_{k}\right)+\sigma \dot{\phi }\left(t_{k}\right)+\frac{\sigma^{2}}{2!}\ddot{\phi }\left(t_{k}\right)+\frac{\sigma^{3}}{3!}\overset{\mathrm{...}}{\phi }\left(t_{k}\right)+O\left(\sigma^{4}\right), \end{array}$$

(16) $$\begin{array}{l} \phi \left(t_{k-1}\right)=\phi \left(t_{k}\right)-\sigma \dot{\phi }\left(t_{k}\right)+\frac{\sigma^{2}}{2!}\ddot{\phi }\left(t_{k}\right)-\frac{\sigma^{3}}{3!}\overset{\mathrm{...}}{\phi }\left(t_{k}\right)+O\left(\sigma^{4}\right), \end{array}$$

(17) $$\begin{array}{l} \phi \left(t_{k-2}\right)=\phi \left(t_{k}\right)-2\sigma \dot{\phi }\left(t_{k}\right)+\frac{4\sigma^{2}}{2!}\ddot{\phi }\left(t_{k}\right)-\frac{8\sigma^{3}}{3!}\overset{\mathrm{...}}{\phi }\left(t_{k}\right)+O\left(\sigma^{4}\right), \end{array}$$

(18) $$\begin{array}{l} \phi \left(t_{k-3}\right)=\phi \left(t_{k}\right)-3\sigma \dot{\phi }\left(t_{k}\right)+\frac{9\sigma^{2}}{2!}\ddot{\phi }\left(t_{k}\right)-\frac{27\sigma^{3}}{3!}\overset{\mathrm{...}}{\phi }\left(t_{k}\right)+O\left(\sigma^{4}\right), \end{array}$$

where the symbol ! denotes the factorial operator, and $\dot{\phi }\left(t_{k}\right)$, $\ddot{\phi }\left(t_{k}\right)$, and $\overset{\mathrm{...}}{\phi }\left(t_{k}\right)$ denote the first-, second-, and third-order derivatives of ϕ(*t*) at time *t*_*k*_, respectively.

Using “36 × (15) − 18 × (16)−27 × (17) + 10 × (18)” yields the following expression:


$$\begin{array}{l} 36\phi \left(t_{k+1}\right)-18\phi \left(t_{k-1}\right)-27\phi \left(t_{k-2}\right)+10\phi \left(t_{k-3}\right)=\phi \left(t_{k}\right)\\ \;+78\sigma \dot{\phi }\left(t_{k}\right)+O\left(\sigma^{4}\right), \end{array}$$

of which the reformulation is given by


(19) $$\begin{array}{l} \dot{\phi }\left(t_{k}\right)=\frac{36\phi \left(t_{k+1}\right)-\phi \left(t_{k}\right)-18\phi \left(t_{k-1}\right)-27\phi \left(t_{k-2}\right)+10\phi \left(t_{k-3}\right)}{78\sigma }\\ \;+O\left(\sigma^{3}\right). \end{array}$$

The expression (Equation 19) is exactly the Taylor difference formula (Equation 14). The proof is thus completed.       ■

Theorem 1 shows that the new Taylor difference formula (Equation 14) is constructed for first-order derivative approximation, and it further indicates that (Equation 14) has a truncation error of *O*(σ^3^).

*Remark 1:* On the one hand, the new Taylor difference formula (Equation 14) has a smaller truncation error than the Euler difference formula (Equation 4), that is, *O*(σ^3^) vs. *O*(σ). Then, the DTZNN model derived by Equation 14 can exhibit a better computational performance than the DTZNN model derived by Equation 4 in solving the TVNE (Equation 1) under the same condition (which can be found in Section 5). On the other hand, though the mathematical derivation is similar (i.e., via Taylor series expansion), the new Taylor difference formula (Equation 14) has a different structure from those formulas (Equation 8, 9, 10), thereby possessing different truncation errors. By summarizing the Taylor difference formulas (Equation 8, 9, 10, 14), the link between Taylor series expansion and numerical differentiation can be obtained. The corresponding DTZNN models with great computational performances are established to solve the TVNE (Equation 1) by utilizing such difference formulas (see Sections 2 and 4). This finding can offer the possibility of developing more DTZNN models to solve different time-varying mathematical problems (Zeng et al., 2024; Zhang and Yi, 2011).

### 3.2 Vector- and matrix-valued forms

Many continuous-time models are depicted in vector- or matrix-valued forms. Thus, extending the new Taylor difference formula (Equation 14) in Theorem 1 to the vector- and matrix-valued forms is necessary.

Specifically, with regard to the approximation of a vector ${\dot{\text{x}}}_{k}=\dot{\text{x}}\left(t_{k}=k\sigma \right)\in {\mathbb{R}}^{n}$, (Equation 14) is reformulated as follows:


(20) $$\begin{array}{l} {\dot{\text{x}}}_{k}=\frac{36{\text{x}}_{k+1}-{\text{x}}_{k}-18{\text{x}}_{k-1}-27{\text{x}}_{k-2}+10{\text{x}}_{k-3}}{78\sigma }+\text{O}\left(\sigma^{3}\right), \end{array}$$

where **O**(σ^3^) ∈ ℝ^*n*^ denotes the vector with every element as *O*(σ^3^). With regard to the approximation of a matrix ${\dot{M}}_{k}=\dot{M}\left(t_{k}=k\sigma \right)\in {\mathbb{R}}^{n\times n}$, Equation 14 is reformulated as follows:


(21) $$\begin{array}{l} {\dot{M}}_{k}=\frac{36M_{k+1}-M_{k}-18M_{k-1}-27M_{k-2}+10M_{k-3}}{78\sigma }+\text{O}\left(\sigma^{3}\right), \end{array}$$

where **O**(σ^3^) ∈ ℝ^*n*×*n*^ denotes the matrix with every element as *O*(σ^3^).

Evidently, each of the difference formulas (Equations 20, 21) has the truncation error of **O**(σ^3^) and is better than the conventional difference formulas (Mathews and Fink, 2004). Therefore, on the one hand, Equation 20 is used to discretize the CTZNN model (Equation 3) for solving the TVNE (Equation 1). On the other hand, by following Guo et al. (2017), Equation 21 is used to establish another DTZNN model for Jacobian matrix inversion. Based on the combination of such two models, the novel CDTZNN model is developed for solving (Equation 1) in this study.

## 4 Novel CDTZNN model

This section proposes the novel CDTZNN model to solve the TVNE (Equation 1) and analyzes the proposed CDTZNN model theoretically.

### 4.1 Model formulation

Discretizing the CTZNN model (Equation 3) via the difference formula (Equation 20) leads to the following expression:


(22) $$\begin{array}{l} {\text{x}}_{k+1}=\frac{1}{36}{\text{x}}_{k}+\frac{1}{2}{\text{x}}_{k-1}+\frac{3}{4}{\text{x}}_{k-2}-\frac{5}{18}{\text{x}}_{k-3}\\ \;-J^{-}\left({\text{x}}_{k},t_{k}\right)(\frac{13}{6}\sigma {\text{f}}_{t}\left({\text{x}}_{k},t_{k}\right)+h\text{f}\left({\text{x}}_{k},t_{k}\right))+\text{O}\left(\sigma^{4}\right), \end{array}$$

where step size *h* = 13γσ/6 ∈ ℝ with γ defined as in Equation 3, and **O**(σ^4^) ∈ ℝ^*n*^ denotes the vector with every element as *O*(σ^4^). By eliminating **O**(σ^4^) from Equation 22, the following DTZNN model for solving the TVNE (Equation 1) is obtained:


(23) $$\begin{array}{l} {\text{x}}_{k+1}=\frac{1}{36}{\text{x}}_{k}+\frac{1}{2}{\text{x}}_{k-1}+\frac{3}{4}{\text{x}}_{k-2}-\frac{5}{18}{\text{x}}_{k-3}\\ \;-J^{-}\left({\text{x}}_{k},t_{k}\right)(\frac{13}{6}\sigma {\text{f}}_{t}\left({\text{x}}_{k},t_{k}\right)+h\text{f}\left({\text{x}}_{k},t_{k}\right))), \end{array}$$

of which the truncation error is **O**(σ^4^).

Furthermore, to avoid the calculation for $J^{-}\left({\text{x}}_{k},t_{k}\right)$ in Equation 23, another DTZNN model for Jacobian matrix inversion is established on the basis of Guo et al. (2017). Such a model is given by


(24) $$\begin{array}{l} M_{k+1}=\frac{1}{36}M_{k}+\frac{1}{2}M_{k-1}+\frac{3}{4}M_{k-2}-\frac{5}{18}M_{k-3}\\ \;-\frac{13}{6}\sigma M_{k}\dot{J}\left({\dot{\text{x}}}_{k},{\text{x}}_{k},t_{k}\right)M_{k}-hM_{k}(J\left({\text{x}}_{k},t_{k}\right)M_{k}-I), \end{array}$$

where *M*_*k*_ denotes the state matrix that can converge to $J^{-}\left({\text{x}}_{k},t_{k}\right)$ with *k* being sufficiently large (which will be presented in Section 5), $\dot{J}\left({\dot{\text{x}}}_{k},{\text{x}}_{k},t_{k}\right)\in {\text{R}}^{n\times n}$ denotes the time derivative of *J*(**x**_*k*_, *t*_*k*_), and *I* ∈ ℝ^*n*×*n*^ denotes the identity matrix. Notably, ${\dot{\text{x}}}_{k}$ is required for calculating $\dot{J}\left({\dot{\text{x}}}_{k},{\text{x}}_{k},t_{k}\right)$, and ${\dot{\text{x}}}_{k}$ is determined by ${\dot{\text{x}}}_{k}=\text{f}\left({\text{x}}_{k},t_{k}\right)+{\text{f}}_{t}\left({\text{x}}_{k},t_{k}\right)$. For completeness, the derivation of the DTZNN model (Equation 24) is presented in the Appendix, which also indicates that Equation 24 has a truncation error of **O**(σ^4^).

Therefore, replacing $J^{-}\left({\text{x}}_{k},t_{k}\right)$ in Equation 23 with *M*_*k*_ and combining Equation 23 with Equation 24 yield the expression as follows:


(25) $$\begin{array}{l} {\text{x}}_{k+1}=\frac{1}{36}{\text{x}}_{k}+\frac{1}{2}{\text{x}}_{k-1}+\frac{3}{4}{\text{x}}_{k-2}-\frac{5}{18}{\text{x}}_{k-3}\\ \;-M_{k}(\frac{13}{6}\sigma {\text{f}}_{t}\left({\text{x}}_{k},t_{k}\right)+h\text{f}\left({\text{x}}_{k},t_{k}\right)),\\ M_{k+1}=\frac{1}{36}M_{k}+\frac{1}{2}M_{k-1}+\frac{3}{4}M_{k-2}-\frac{5}{18}M_{k-3}\\ \;-\frac{13}{6}\sigma M_{k}\dot{J}\left({\dot{\text{x}}}_{k},{\text{x}}_{k},t_{k}\right)M_{k}-hM_{k}(J\left({\text{x}}_{k},t_{k}\right)M_{k}-I), \end{array}$$

which is the proposed CDTZNN model to solve the TVNE (Equation 1). Evidently, the proposed CDTZNN model (Equation 25) does not require calculating $J^{-}\left({\text{x}}_{k},t_{k}\right)$ at each iteration. Thus, the model can reduce the amount of calculation.

**Table:** 

**Algorithm**	**Procedure of CDTZNN model (Equation 25) for solving TVNE (Equation 1)**
Step 1: Initialization	Set up time duration *T*, sampling gap σ, and step size *h*. Initialize *t*_0_, **x**_0_, and *M*_0_. Receive **f**(**x**_0_, *t*_0_), **f**_*t*_(**x**_0_, *t*_0_), and *J*(**x**_0_, *t*_0_). Compute ||**e**_0_||_2_ = ||**f**(**x**_0_, *t*_0_)||_2_.
Step 2: First Loop (*k* = 0, 1, 2)	Compute ${\dot{\text{x}}}_{k}=\text{f}\left({\text{x}}_{k},t_{k}\right)+{\text{f}}_{t}\left({\text{x}}_{k},t_{k}\right)$, and $\dot{J}\left({\dot{\text{x}}}_{k},{\text{x}}_{k},t_{k}\right)$. Compute **x**_*k*+1_ and *M*_*k*+1_ through (Equation 26). Receive **f**(**x**_*k*+1_, *t*_*k*+1_), **f**_*t*_(**x**_*k*+1_, *t*_*k*+1_), and *J*(**x**_*k*+1_, *t*_*k*+1_). Compute ||**e**_*k*+1_||_2_ = ||**f**(**x**_*k*+1_, *t*_*k*+1_)||_2_.
Step 3: Second loop (*k* = 3, ⋯ , *T*/σ)	Compute ${\dot{\text{x}}}_{k}=\text{f}\left({\text{x}}_{k},t_{k}\right)+{\text{f}}_{t}\left({\text{x}}_{k},t_{k}\right)$, and $\dot{J}\left({\dot{\text{x}}}_{k},{\text{x}}_{k},t_{k}\right)$. Compute **x**_*k*+1_ and *M*_*k*+1_ through (Equation 25). Receive **f**(**x**_*k*+1_, *t*_*k*+1_), **f**_*t*_(**x**_*k*+1_, *t*_*k*+1_), and *J*(**x**_*k*+1_, *t*_*k*+1_). Compute ||**e**_*k*+1_||_2_ = ||**f**(**x**_*k*+1_, *t*_*k*+1_)||_2_.
Step 4: Output	Save state vector **x**_*k*_ and computational error ||**e**_*k*_||_2_. Plot the corresponding figures.

For the proposed CDTZNN model (Equation 25), with the given initial states **x**_0_ and *M*_0_, three states (i.e., **x**_1_, **x**_2_, and **x**_3_) for the first recursion and three states (i.e., *M*_1_, *M*_2_, and *M*_3_) for the second recursion are obtained via the following computation:


(26) $$\begin{array}{l} \{\begin{array}{l} {\text{x}}_{1}={\text{x}}_{0}-M_{0}(\sigma {\text{f}}_{t}\left({\text{x}}_{0},t_{0}\right)+h\text{f}\left({\text{x}}_{0},t_{0}\right)),\\ M_{1}=M_{0}-\sigma M_{0}\dot{J}\left({\dot{\text{x}}}_{0},{\text{x}}_{0},t_{0}\right)M_{0}-hM_{0}(J\left({\text{x}}_{0},t_{0}\right)M_{0}-I),\\ {\text{x}}_{2}={\text{x}}_{1}-M_{1}(\sigma {\text{f}}_{t}\left({\text{x}}_{1},t_{1}\right)+h\text{f}\left({\text{x}}_{1},t_{1}\right)),\\ M_{2}=M_{1}-\sigma M_{1}\dot{J}\left({\dot{\text{x}}}_{1},{\text{x}}_{1},t_{1}\right)M_{1}-hM_{1}(J\left({\text{x}}_{1},t_{1}\right)M_{1}-I),\\ {\text{x}}_{3}={\text{x}}_{2}-M_{2}(\sigma {\text{f}}_{t}\left({\text{x}}_{2},t_{2}\right)+h\text{f}\left({\text{x}}_{2},t_{2}\right)),\\ M_{3}=M_{2}-\sigma M_{2}\dot{J}\left({\dot{\text{x}}}_{2},{\text{x}}_{2},t_{2}\right)M_{2}-hM_{2}(J\left({\text{x}}_{2},t_{2}\right)M_{2}-I). \end{array} \end{array}$$

In addition, for better understanding, the procedure of using the proposed CDTZNN model (Equation 25) to solve the TVNE (Equation 1) is presented in the Algorithm.

### 4.2 Theoretical analysis

This subsection theoretically analyzes the proposed CDTZNN model (Equation 25) to solve the TVNE (Equation 1) via the following theorems.

**Theorem 2**. *The proposed CDTZNN model (**Equation 25**) is zero stable and consistent and thus possesses the convergence property*.

**Proof**. For the proposed CDTZNN model (Equation 25), the characteristic polynomials of the first and second recursions are the same as each other, which are written as the following unified form:


$$\begin{array}{l} {\tilde{P}}_{1}\left(\vartheta \right)=\vartheta^{4}-\frac{1}{36}\vartheta^{3}-\frac{1}{2}\vartheta^{2}-\frac{3}{4}\vartheta +\frac{5}{18}. \end{array}$$

The roots of ${\tilde{P}}_{1}\left(\vartheta \right)=0$ are given by


$$\begin{array}{l} \vartheta_{1}=-0.5211+i0.4694,\\ \vartheta_{2}=-0.5211-i0.4694,\\ \vartheta_{3}=0.2372+i0.8065,\\ \vartheta_{4}=0.2372-i0.8065, \end{array}$$

where *i* denotes the imaginary unit. All the roots lie in the unit disk. According to Griffiths and Higham (2010), Equation 25 is zero stable.

On the basis of Section 4.1, the DTZNN models (Equation 23, 24) have the truncation error of **O**(σ^4^). With the combination of such two models, the proposed CDTZNN model (Equation 25) is derived. This statement shows that the truncation error of (Equation 25) is **O**(σ^4^). According to Griffiths and Higham (2010), the proposed CDTZNN model (Equation 25) is consistent. The zero stability and consistency ensure the convergence property of Equation 25. The proof is thus completed.       ■

**Theorem 3**. *The proposed CDTZNN model (**Equation 25**) is a linear convergent method for solving the TVNE (**Equation 1**)*.

**Proof**. Let Δ**x**_*k*_ = **x**_*k*+1_−**x**_*k*_ and ${\text{x}}_{k}^{*}={\text{x}}^{*}\left(t_{k}=k\tau \right)$ be the theoretical solution of Equation 1 (i.e., $\text{f}\left({\text{x}}_{k}^{*},t_{k}\right)=0$). Considering the truncation error, the first recursion of the proposed CDTZNN model (Equation 25) is rewritten as follows:


$$\begin{array}{l} \Delta {\text{x}}_{k}=-\frac{35}{36}\Delta {\text{x}}_{k-1}-\frac{17}{36}\Delta {\text{x}}_{k-2}+\frac{5}{18}\Delta {\text{x}}_{k-3}\\ \;-M_{k}(\frac{13}{6}\sigma {\text{f}}_{t}\left({\text{x}}_{k},t_{k}\right)+h\text{f}\left({\text{x}}_{k},t_{k}\right))+\text{O}\left(\sigma^{4}\right), \end{array}$$

which is equivalent to the following expression:


(27) $$\begin{array}{l} \Delta {\text{x}}_{k}=\frac{7}{13}\Delta {\text{x}}_{k}-\frac{35}{78}\Delta {\text{x}}_{k-1}-\frac{17}{78}\Delta {\text{x}}_{k-2}+\frac{5}{39}\Delta {\text{x}}_{k-3}\\ \;-M_{k}(\sigma {\text{f}}_{t}\left({\text{x}}_{k},t_{k}\right)+\frac{6}{13}h\text{f}\left({\text{x}}_{k},t_{k}\right))+\text{O}\left(\sigma^{4}\right). \end{array}$$

It follows from Taylor series expansion (Mathews and Fink, 2004) that


(28) $$\begin{array}{l} \text{f}\left({\text{x}}_{k+1},t_{k+1}\right)=\text{f}\left({\text{x}}_{k}+\Delta {\text{x}}_{k},t_{k}+\sigma \right)\\ \;=\text{f}\left({\text{x}}_{k},t_{k}\right)+J\left({\text{x}}_{k},t_{k}\right)\Delta {\text{x}}_{k}+\sigma {\text{f}}_{t}\left({\text{x}}_{k},t_{k}\right)+\text{O}\left(\sigma^{2}\right), \end{array}$$

with $\text{O}\left(∥\Delta {\text{x}}_{k}{∥}_{2}^{2}\right)$ being absorbed into **O**(σ^2^). Let us assume that *M*_*k*_ computed by the second recursion of Equation 25 is exactly equal to $J^{-}\left({\text{x}}_{k},t_{k}\right)$, that is, satisfying *J*(**x**_*k*_, *t*_*k*_)*M*_*k*_ − *I*. Then, substituting Equation 27 into Equation 28 yields


(29) $$\begin{array}{l} \text{f}\left({\text{x}}_{k+1},t_{k+1}\right)=J\left({\text{x}}_{k},t_{k}\right)\left(\frac{7}{13}\Delta {\text{x}}_{k}-\frac{35}{78}\Delta {\text{x}}_{k-1}-\frac{17}{78}\Delta {\text{x}}_{k-2}+\frac{5}{39}\Delta {\text{x}}_{k-3}\right)\\ \;+\left(1-\frac{6}{13}h\right)\text{f}\left({\text{x}}_{k},t_{k}\right)+\text{O}\left(\sigma^{2}\right)+\text{O}\left(\sigma^{4}\right). \end{array}$$

Notably, Theorem 2 and its proof indicate that **x**_*k*_ computed by Equation 25 would converge to ${\text{x}}_{k}^{*}$. In addition, when *k* is sufficiently large, ${\text{x}}_{k}={\text{x}}_{k}^{*}+\text{O}\left(\sigma^{4}\right)$, and $\text{f}\left({\text{x}}_{k},t_{k}\right)=\text{f}\left({\text{x}}_{k}^{*},t_{k}\right)+\text{O}\left(\sigma^{4}\right)$. In view of that $\text{f}\left({\text{x}}_{k}^{*},t_{k}\right)=0$, $J\left({\text{x}}_{k},t_{k}\right)\left(42\Delta {\text{x}}_{k}-35\Delta {\text{x}}_{k-1}-17\Delta {\text{x}}_{k-2}+10\Delta {\text{x}}_{k-3}\right)/78+\text{O}\left(\sigma^{2}\right)=\alpha \text{f}\left({\text{x}}_{k},t_{k}\right)+\text{O}\left(\sigma^{4}\right)$, with α ∈ ℝ denoting the correction parameter and *k* being sufficiently large. Thus, the reformulation of Equation 29 is given by


(30) $$\begin{array}{l} \text{f}\left({\text{x}}_{k+1},t_{k+1}\right)=\left(1-\frac{6}{13}h+\alpha \right)\text{f}\left({\text{x}}_{k},t_{k}\right)+\text{O}\left(\sigma^{4}\right). \end{array}$$

The error function **e**(*t*) defined in Equation 2 is recalled, and the following result is obtained from Equation 30:


(31) $$\begin{array}{l} {\text{e}}_{k}=\text{f}\left({\text{x}}_{k},t_{k}\right)=\left(1-\frac{6}{13}h+\alpha \right){\text{e}}_{k-1}+\text{O}\left(\sigma^{4}\right)\\ \;=\left(1-\frac{6}{13}h+\alpha \right)\left(\left(1-\frac{6}{13}h+\alpha \right){\text{e}}_{k-2}+\text{O}\left(\sigma^{4}\right)\right)+\text{O}\left(\sigma^{4}\right)\\ \;={\left(1-\frac{6}{13}h+\alpha \right)}^{2}{\text{e}}_{k-2}+\left(1-\frac{6}{13}h+\alpha \right)\text{O}\left(\sigma^{4}\right)+\text{O}\left(\sigma^{4}\right)\\ \;\vdots \\ \;={\left(1-\frac{6}{13}h+\alpha \right)}^{k}{\text{e}}_{0}+\beta \text{O}\left(\sigma^{4}\right), \end{array}$$

where ${\text{e}}_{0}\in {\mathbb{R}}^{n}$ denotes the initial error and β > 0 ∈ ℝ denotes a constant. As presented in Theorem 2, the proposed CDTZNN model (Equation 25) possesses the convergence property, which means that the error function **e**_*k*_ is convergent. With regard to Equation 31, the convergence of **e**_*k*_ implies that −1 < 1 − 6*h*/13+α < 1. Therefore, on the basis of Equation 30,


(32) $$\begin{array}{l} \underset{k\to \infty }{\lim}\frac{∥{\text{e}}_{k+1}{∥}_{2}}{∥{\text{e}}_{k}{∥}_{2}}=|1-\frac{6}{13}h+\alpha |<1, \end{array}$$

which shows the linear convergence of the sequence {**x**_*k*_} computed by Equation 25. Thus, the proposed CDTZNN model (Equation 25) is a linear convergent method for solving the TVNE (Equation 1, which completes the proof.       ■

**Theorem 4**. *When a solvable TVNE (**Equation 1**) is considered, the SSCE of the proposed CDTZNN model (**Equation 25**) is of* *O*(σ^4^).

**Proof**. Theorem 3 and its proof indicate that −1 < 1 − 6*h*/13+α < 1. Then, $\lim_{k\to \infty }{\left(1-6h/13+\alpha \right)}^{k}=0$. Therefore, the following result is obtained:


$$\begin{array}{l} \underset{k\to \infty }{\lim}∥{\text{e}}_{k}{∥}_{2}=\underset{k\to \infty }{\lim}∥\text{f}\left({\text{x}}_{k},t_{k}\right){∥}_{2}=\underset{k\to \infty }{\lim}∥\beta \text{O}\left(\sigma^{4}\right){∥}_{2}=O\left(\sigma^{4}\right), \end{array}$$

which indicates that the SSCE of the proposed CDTZNN model (Equation 25) is of *O*(σ^4^). The proof is thus completed.       ■

In summary, Theorems 2 through 4 theoretically guarantee the computational performance of Equation 25 in solving the TVNE (Equation 1). That is, the proposed CDTZNN model (Equation 25) can effectively generate an exact solution of Equation 1)

## 5 Numerical verification and robot application

In this section, comparative numerical experiments are conducted to demonstrate the efficacy of the proposed CDTZNN model (Equation 25). Moreover, such a model is applied to the motion planning of robot manipulators to show the applicability of CDTZNN.

### 5.1 Model comparison

This subsection shows the comparative results of using the Broyden-aided DTZNN model (Equation 7) and the proposed CDTZNN model (Equation 25) to solve the following TVNE:


(33) $$\begin{array}{l} \text{f}\left(\text{x}\left(t\right),t\right)=\left[\begin{array}{c} \ln\left(x_{1}\left(t\right)\right)-1/\left(t+1\right)\\ x_{1}\left(t\right)x_{2}\left(t\right)-\sin\left(t\right)\exp\left(1/\left(t+1\right)\right)\\ x_{1}^{2}\left(t\right)-\sin\left(t\right)x_{2}\left(t\right)+x_{3}\left(t\right)-2\\ x_{1}^{2}\left(t\right)-x_{2}^{2}\left(t\right)+x_{3}\left(t\right)+x_{4}\left(t\right)-t \end{array}\right]=0. \end{array}$$

**Figure 1:**
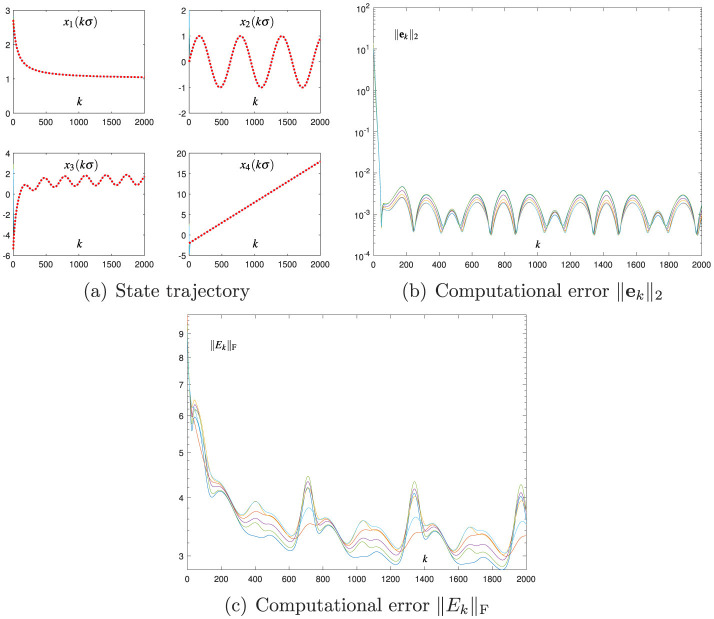
Numerical results of using the Broyden-aided DTZNN model (Equation 7) with σ = 0.01 and *h* = 0.2 for solving the TVNE (Equation 33). **(A)** State trajectory. **(B)** Computational error ||**e**_*k*_||_2_. **(C)** Computational error ||*E*_*k*_||_F_.

**Figure 2:**
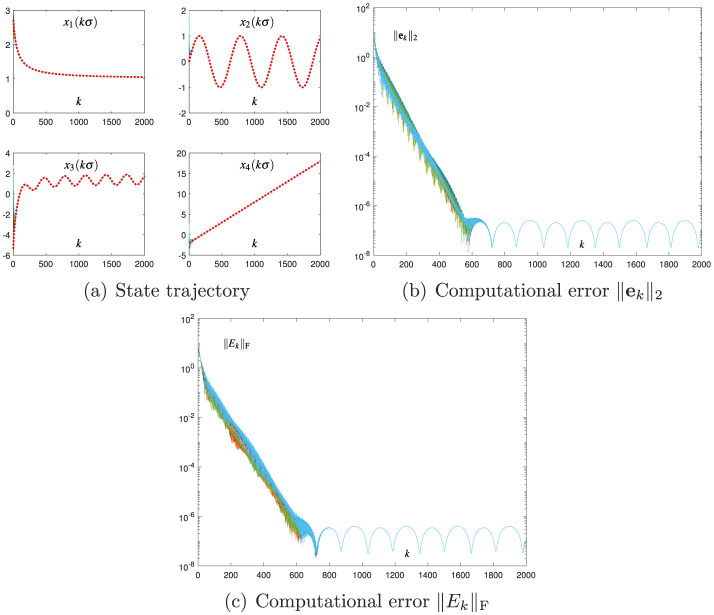
Numerical results of using the proposed CDTZNN model (Equation 25) with σ = 0.01 and *h* = 0.2 for solving the TVNE (Equation 33). **(A)** State trajectory. **(B)** Computational error ||**e**_*k*_||_2_. **(C)** Computational error ||*E*_*k*_||_F_.

**Figure 3:**
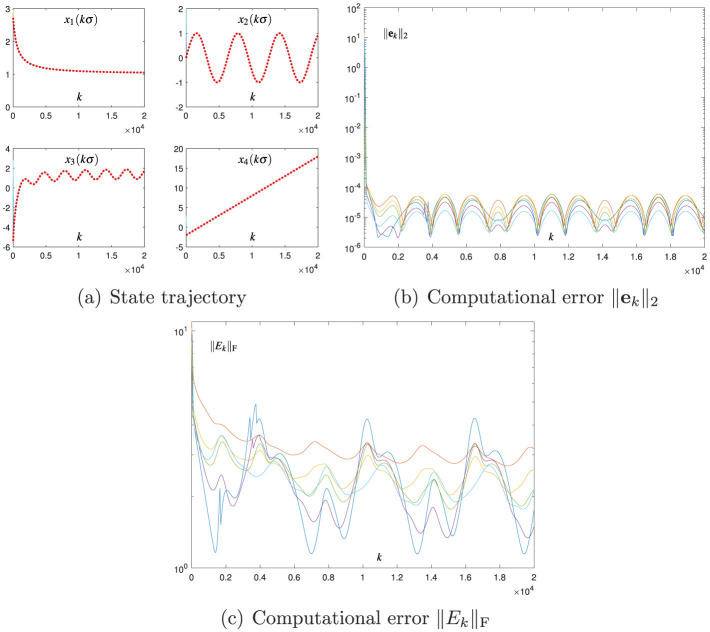
Numerical results of using the Broyden-aided DTZNN model (Equation 7) with σ = 0.001 and *h* = 0.2 for solving the TVNE (Equation 33). **(A)** State trajectory. **(B)** Computational error ||**e**_*k*_||_2_. **(C)** Computational error ||*E*_*k*_||_F_.

**Figure 4:**
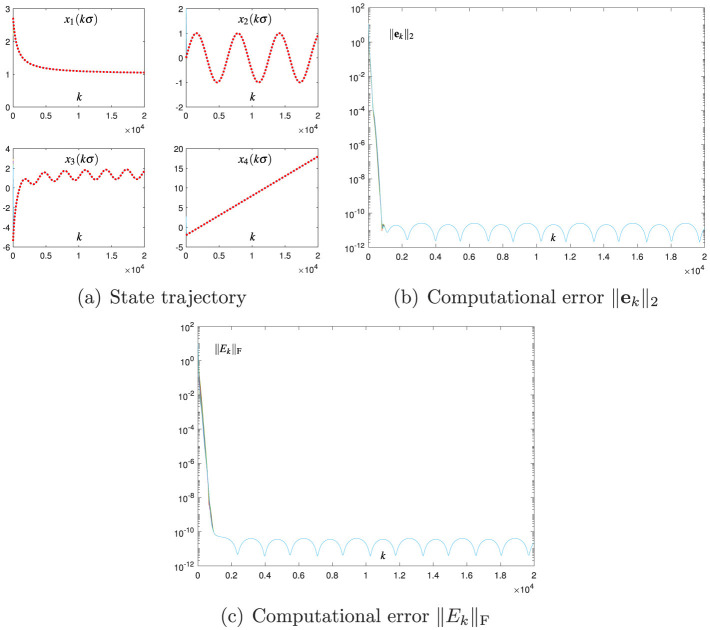
Numerical results of using the proposed CDTZNN model (Equation 25) with σ = 0.001 and *h* = 0.2 for solving the TVNE (Equation 33). **(A)** State trajectory. **(B)** Computational error ||**e**_*k*_||_2_. **(C)** Computational error ||*E*_*k*_||_F_.

The theoretical solution to Equation 33 is


$$\begin{array}{l} {\text{x}}^{*}\left(t\right)=\left[\begin{array}{c} \exp\left(1/\left(t+1\right)\right)\\ \sin\left(t\right)\\ 2-\exp\left(2/\left(t+1\right)\right)+\sin^{2}\left(t\right)\\ t-2 \end{array}\right], \end{array}$$

which is given to validate the effectiveness of Equations 7, 25. Notably, the DTZNN models (Equations 5, 12) for solving Equation 33 have been investigated and compared in Guo et al. (2018), and their studies are thus omitted in this paper. The numerical results using Equations 7, 25 to solve Equation 33 are provided in Figures 1–4.

Figure 1 presents the results of using the Broyden-aided DTZNN model (Equation 7) with σ = 0.01 and *h* = 0.2, where the computational error ||*E*_*k*_||_F_ is obtained by ||*E*_*k*_||_F_ = ||*J*(**x**_*k*_, *t*_*k*_)*M*_*k*_ − *I*||_F_ with ||·||_F_ denoting the Frobenius norm of a matrix. As presented in Figure 1a, each state vector **x**_*k*_ of Equation 7 converges to the theoretical solution of Equation 33, that is, ${\text{x}}_{k}\to {\text{x}}_{k}^{*}={\text{x}}^{*}\left(t=k\sigma \right)$ as *k* evolves. As presented in Figure 1b, each computational error ||**e**_*k*_||_2_ of Equation 7 presents the convergence characteristic, and the corresponding SSCE is of the order 10^−3^. Notably, Figure 1c shows the computational error ||*E*_*k*_||_F_ via the Broyden method (Equation 6) during the process of solving (Equation 33) for illustration and investigation. As shown in Figure 1c, ||*E*_*k*_||_F_ at steady state is of the order 10^−3^. These results indicate that the Broyden method (Equation 6) is effectively utilized to replace $J^{-}\left({\text{x}}_{k},t_{k}\right)$ in Equation 5, and they verify that the Broyden-aided DTZNN model (Equation 7) can effectively solve the TVNE (Equation 33).

Figure 2 shows the results of using the proposed CDTZNN model (Equation 25) with σ = 0.01 and *h* = 0.2. As shown in Figure 2a, each state vector **x**_*k*_ of Equation 25 is convergent to ${\text{x}}_{k}^{*}$ of Equation 33. As shown in Figure 2b, each computational error ||**e**_*k*_||_2_ of the first recursion in Equation 25 also shows the convergence characteristic, and the corresponding SSCE is of the order 10^−7^. As shown in Figure 2c, each computational error ||*E*_*k*_||_F_ of the second recursion in Equation 25 at steady state is of the order 10^−7^. These results verify that Equation 24 is successfully combined with Equation 23, and they substantiate that Equation 25 can effectively solve Equation 33. The comparison of Figure 1b with Figure 2b shows that the SSCE of Equation 25 is approximately 1, 000 times smaller than the SSCE of Equation 7. In addition, the comparison of Figure 1c with Figure 2c reveals that the DTZNN model (Equation 24) is more effective than the Broyden method (Equation 6) in replacing $J^{-}\left({\text{x}}_{k},t_{k}\right)$ at each iteration. These comparative results indicate that the proposed CDTZNN model (Equation 25) is advantageous over the Broyden-aided DTZNN model (Equation 7) in solving the TVNE (Equation 33).

The Broyden-aided DTZNN model (Equation 7) and the proposed CDTZNN model (Equation 25) are studied by decreasing σ from 0.01 to 0.001 and fixing the value of *h*. The related numerical results are presented in Figures 3 and 4, which demonstrate the effectiveness of Equation 7, 25 in terms of the state vector **x**_*k*_ converging to ${\text{x}}_{k}^{*}$ and the computational errors ||**e**_*k*_||_2_ and ||*E*_*k*_||_F_ being sufficiently small. In particular, by observing Figures 1–4, the decrease in σ leads to the decrease in SSCEs. In addition, by decreasing σ, the performance of Equation 25 is improved more effectively than that of Equation 7. That is, when σ decreases by 10 times, ||**e**_*k*_||_2_ of Equation 25 at steady state decreases by 10, 000 times (i.e., from 10^−7^ to 10^−11^), and ||**e**_*k*_||_2_ of Equation 7 at steady state decreases only by 100 times (i.e., from 10^−3^ to 10^−5^). Therefore, the proposed CDTZNN model (Equation 25) is superior to the Broyden-aided DTZNN model (Equation 7).

The abovementioned numerical results in Figures 1–4 substantiate the efficacy of the proposed CDTZNN model (Equation 25) in solving the TVNE (Equation 33).

### 5.2 Effect of σ and *h*

In this subsection, the effect of σ and *h* on six different DTZNN models (Equations 5, 7, 11, 12, 13, 25) is studied. Table 1 shows the corresponding numerical results, where the initial state is ${\text{x}}_{0}=0.2\in {\mathbb{R}}^{4}$.

**Table 1:** SSCE of six different DTZNN models (Equation 5, 7, 11, 12, 13, 25) with different σ and *h* for solving the TVNE (Equation 33).

**#**	***h***	**σ = 0.01**	**σ = 0.001**	**Mode**
Euler DTZNN (Equation 5)	0.10	1.399 × 10^−3^	1.414 × 10^−5^	*O*(σ^2^)
	0.15	9.385 × 10^−4^	9.422 × 10^−6^	
	0.20	7.056 × 10^−4^	7.070 × 10^−6^	
Broyden-aided DTZNN (Equation 7)	0.10	3.660 × 10^−3^	4.616 × 10^−5^	*O*(σ^2^)
	0.15	2.397 × 10^−3^	3.066 × 10^−5^	
	0.20	2.109 × 10^−3^	2.927 × 10^−5^	
Taylor DTZNN-I (Equation 11)	0.10	4.554 × 10^−7^	4.945 × 10^−11^	*O*(σ^4^)
	0.15	3.173 × 10^−7^	3.299 × 10^−11^	
	0.20	2.420 × 10^−7^	2.474 × 10^−11^	
Taylor DTZNN-II (Equation 12)	0.10	6.612 × 10^−7^	7.059 × 10^−11^	*O*(σ^4^)
	0.15	4.569 × 10^−7^	4.712 × 10^−11^	
	0.20	3.472 × 10^−7^	3.535 × 10^−11^	
Taylor DTZNN-III (Equation 13)	0.10	1.260 × 10^−8^	1.447 × 10^−13^	*O*(σ^5^)
	0.15	8.913 × 10^−9^	9.763 × 10^−14^	
	0.20	6.837 × 10^−8^	7.618 × 10^−14^	
CDTZNN (Equation 25)	0.10	4.874 × 10^−7^	5.184 × 10^−11^	*O*(σ^4^)
	0.15	3.362 × 10^−7^	3.456 × 10^−11^	
	0.20	2.554 × 10^−7^	2.592 × 10^−11^	

It follows from Table 1 that the computational errors ||**e**_*k*_||_2_ at steady state, i.e., SSCE, are sufficiently small, and the effectiveness of Equations 5, 7, 11, 12, 13, 25) in solving Equation 33 is thus validated. By observing the numerical results in Table 1, the following summary is obtained.

1) The computational performance of four different DTZNN models (Equations 5, 7, 12, 25) is improved by decreasing the value of σ, which shows the importance of σ in such models.
2) The SSCE of the Euler DTZNN model (Equation 5) and the Broyden-aided DTZNN model (Equation 7) is expressed in the mode of *O*(σ^2^). That is, the decrease in σ by 10 times results in the decrease in SSCE by 100 times. This phenomenon indicates the *O*(σ^2^) mode of Equations 5 or 7 in solving Equation 33, which coincides with Lemma 2.
3) The SSCEs of the Taylor DTZNN-I model (Equation 11) and the Taylor DTZNN-II model (Equation 12) are expressed in the mode of *O*(σ^4^). That is, the decrease in σ by 10 times results in the decrease in SSCE by 10, 000 times. This phenomenon indicates the *O*(σ^4^) mode of Equation 12 in solving Equation 33, which coincides with Lemma 3.
4) The SSCE of the Taylor DTZNN-III model (Equation 13) is expressed in the mode of *O*(σ^5^). That is, the decrease in σ by 10 times results in the decrease in SSCE by 100, 000 times. This phenomenon indicates the *O*(σ^5^) mode of Equation 12 in solving Equation 33, which coincides with Lemma 3.
5) The SSCE of the proposed CDTZNN model (Equation 25) is also expressed in the mode of *O*(σ^4^). That is, the decrease in σ by 10 times results in the decrease in SSCE by 10, 000 times. This phenomenon indicates the *O*(σ^4^) mode of Equation 25 in solving Equation 33, which coincides with Theorem 4.
6) The computational performance of four different DTZNN models (Equations 5, 7, 11, 12, 13, 25) is further improved in solving Equation 33 with the appropriate increase in *h*.

On the basis of these results, a small value of σ (e.g., σ = 0.01) and a relatively large value *h* (e.g., *h* = 0.2) can ensure the superior computational performance of the proposed CDTZNN model (Equation 25).

*Remark 2:* With regard to the existing DTZNN models (Equation 5, 11, 12, 13) that require calculating $J^{-}\left({\text{x}}_{k},t_{k}\right)$ at each iteration, they have at least the computational complexity of *O*(*n*^3^). In this sense, the proposed CDTZNN model (Equation 25) has the same computational complexity as Equations 5, 11, 12, 13. Notably, the direct calculation of $J^{-}\left({\text{x}}_{k},t_{k}\right)$ is not required in Equation 25, and the amount of calculation can thus be effectively reduced. Compared with the Broyden-aided DTZNN model (Equation 7), more multiplications and more additions are needed for the proposed CDTZNN model (Equation 25). However, Equation 25 has a better performance than Equation 7 in solving Equation 1, which can be concluded by Table 1. In summary, the proposed CDTZNN model (Equation 25) is advantageous over the existing DTZNN models (Equation 5, 7, 11, 12, 13) in solving the TVNE (Equation 1).

### 5.3 Application to robot manipulators

In general, the motion planning of a robot manipulator is described as that the joint configuration **q**(*t*) ∈ ℝ^*n*^ should be determined when giving the end-effector path **r**(*t*) ∈ ℝ^*n*^ (Li et al., 2019). Mathematically, the purpose of the robot's motion planning can be achieved by effectively solving the TVNE as follows:


(34) $$\begin{array}{l} \text{f}\left(\text{q}\left(t\right)\right)=\text{r}\left(t\right), \end{array}$$

where **f**(·) denotes the differentiable nonlinear mapping with known parameters for a specific robot manipulator.

By extending the proposed CDTZNN model (Equation 25) to solve Equation 34, the following expression is obtained:


(35) $$\begin{array}{l} {\text{q}}_{k+1}=\frac{1}{36}{\text{q}}_{k}+\frac{1}{2}{\text{q}}_{k-1}+\frac{3}{4}{\text{q}}_{k-2}-\frac{5}{18}{\text{q}}_{k-3}\\ \;+M_{k}(\frac{13}{6}\sigma {\dot{\text{r}}}_{k}-h\left(\text{f}\left({\text{q}}_{k}\right)-{\text{r}}_{k}\right)),\\ M_{k+1}=\frac{1}{36}M_{k}+\frac{1}{2}M_{k-1}+\frac{3}{4}M_{k-2}-\frac{5}{18}M_{k-3}\\ \;-\frac{13}{6}\sigma M_{k}\dot{J}\left({\dot{\text{q}}}_{k},{\text{q}}_{k}\right)M_{k}-hM_{k}(J\left({\text{x}}_{k}\right)M_{k}-I), \end{array}$$

where the joint configuration **q**_*k*_ = **q**(*t* = *kσ*), and the desired end-effector path **r**_*k*_ = **r**(*t* = *kσ*) with **ṙ**_*k*_ as its time derivative. Therefore, the new motion planning scheme (Equation 35) without need for direct Jacobian matrix inversion is derived for robot manipulators.

It is worth mentioning here that the calculation procedure of the new motion planning scheme (Equation 35) is similar to that in the Algorithm. With the combined initial state {**q**_0_, *M*_0_}, the three other combined states required for Equation 35, namely, {**q**_1_, *M*_1_}, {**q**_2_, *M*_2_}, and {**q**_3_, *M*_3_}, are obtained via the calculation that is similar to Equation 26. Then, on the basis of the iterative calculation in Equation 35, the sequences of joint configuration {**q**_*k*_} are obtained, where *k* = 4, 5, ⋯ , *T*/σ. Given that {**q**_*k*_} is determined, the motion planning of robot manipulators is realized by Equation 35.

The following simulation results under the DOBOT manipulator (Xiao et al., 2019) are presented for validating the new motion planning scheme (Equation 35). In the simulations, the initial DOBOT joint configuration is **q**_0_ = [0;π/6;−π/4] rad, and the motion task duration is *T* = 10 s.

**Figure 5:**
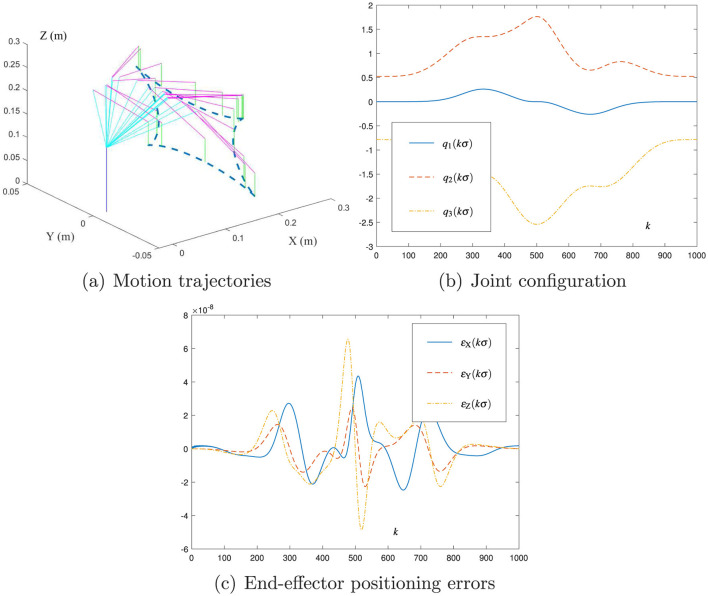
Simulation results of using the new motion planning scheme (Equation 35) with σ = 0.01 and *h* = 0.2 for the DOBOT manipulator to track the tetracuspid path. **(A)** Motion trajectories. **(B)** Joint configuration. **(C)** End-effector positioning errors.

Figure 5 presents the results of using the new motion planning scheme (Equation 35) with σ = 0.01 and *h* = 0.2 for the DOBOT manipulator to track a tetracuspid path, where the end-effector planning error is obtained as $\varepsilon_{k}=\text{f}\left({\text{q}}_{k}\right)-{\text{r}}_{k}\in {\mathbb{R}}^{3}$. As shown in Figures 5a, b, the DOBOT joint configuration {**q**_*k*_} during the duration is determined via (Equation 35). Therefore, the motion planning task is successfully fulfilled. As shown in Figure 5c, the end-effector planning error is of order 10^−8^ m, which means that the DOBOT end-effector trajectory and the desired path match well. Notably, the initial point of the desired path is calculated and confirmed by *f*(**q**_0_), which means that the end-effector planning error ε_*k*_ at the initial time instant, i.e., ε_0_, is zero. Because of intrinsical DOBOT nonlinearity as well as the transient error computed by Equation 35, ε_*k*_ will increase in the transient phase. Since Equation 35 has the property of convergence, the magnitude of ε_*k*_ can be kept within the region of a small value, as presented in Figure 5c. These simulation results substantiate that the new scheme (**Equation 35**) is effective in the motion planning of the DOBOT manipulator.

**Figure 6:**
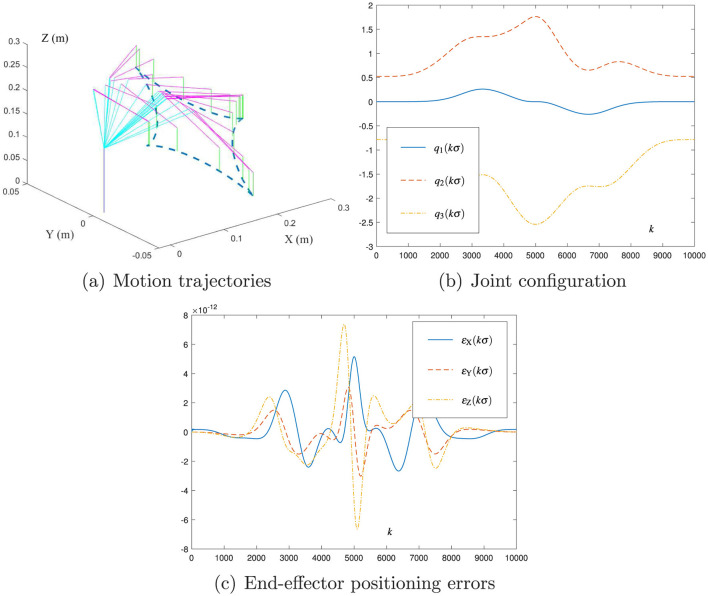
Simulation results of using the new motion planning scheme (Equation 35) with σ = 0.001 and *h* = 0.2 for the DOBOT manipulator to track the tetracuspid path. **(A)** Motion trajectories. **(B)** Joint configuration. **(C)** End-effector positioning errors.

The new motion planning scheme (Equation 35) is tested with the decrease in σ, and Figure 6 presents the related simulation results. As presented in Figure 6, the DOBOT end-effector successfully follows the desired tetracuspid path with the maximal planning error being of order 10^−12^ m, which indicates the effectiveness of Equation 35). Moreover, the comparison of Figure 5c with Figure 6c shows that the decrease in σ by 10 times leads to the decrease in the maximal planning error by 10, 000 times (i.e., from 10^−8^ m to 10^−12^ m). This phenomenon shows the important role of σ in the new motion planning scheme (Equation 35), and it expresses the *O*(σ^4^) form in the planning error via Equation refmp.new. Thus, σ in Equation 35 can be set as a small value to realize the required precision in robot applications.

**Figure 7:**
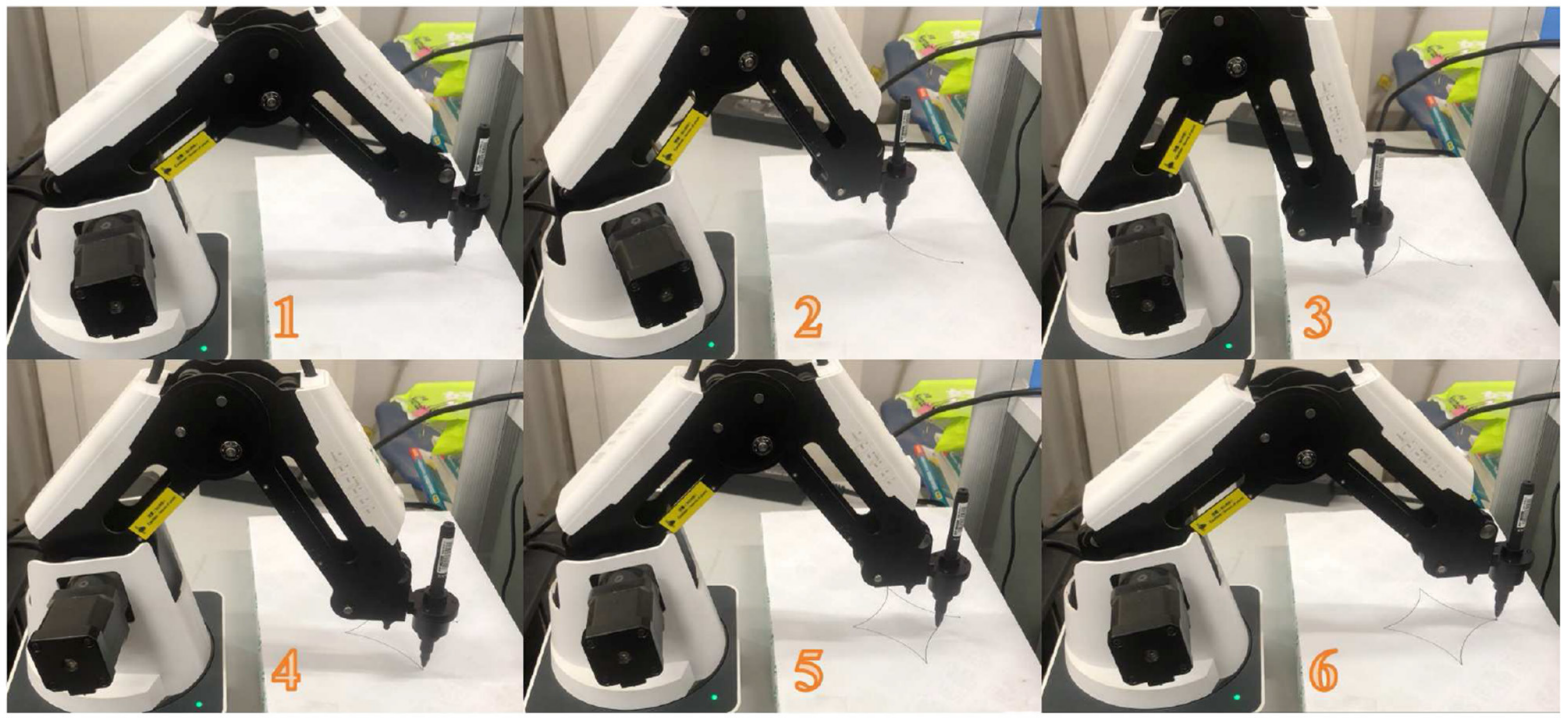
Experiment results of using the new motion planning scheme (Equation 35) with σ = 0.01 and *h* = 0.2 for the DOBOT manipulator to track the tetracuspid path.

Based on these simulation results, the new motion planning scheme (Equation 35) with σ = 0.01 and *h* = 0.2 is applied to the practical DOBOT manipulator, and the related experiment results are shown in Figure 7. Evidently, Figure 7 indicates that the DOBOT end-effector successfully tracks the desired tetracuspid path. Thus, the new motion planning scheme (Equation 35) is implemented effectively on the practical ROBOT manipulator.

*Remark 3:* As indicated by Figures 5, 6, the new motion planning scheme (Equation 35) possesses the *O*(σ^4^) form in the planning error. Then, even the sampling time σ is selected as a large value (e.g., 0.1 s), the robot's planning error can be small enough (may be of order 10^−4^ m). Such a accuracy is still acceptable for robot manipulators. In practice, the sampling time σ of robot manipulators is generally selected to be 0.01s (and/or 0.02 s), as presented in Figure 7. With regard to this value of σ, the robot's planning error is kept within a sufficiently small value (e.g., of order 10^−7^ m or 10^−8^ m). In this sense, the planning accuracy and real-time feasibility can be both guaranteed by the new scheme (Equation 35) for the motion planning of robot manipulators.

In summary, the above simulation and experiment results in Figures 5–7 validate the efficacy of the new motion planning scheme (Equation 35) and show the applicability of the proposed CDTZNN model (Equation 25) to robot manipulators.

## 6 Conclusions

This study proposes a novel CDTZNN model (Equation 25) for solving the TVNE (Equation 1) on the basis of the discretization of the CTZNN model (Equation 3) using a new Taylor difference formula (Equation 14). Such a model is the combination of two DTZNN models (Equation 23) and Equation 24, and it does not require calculating the inverse of Jacobian matrix (thus reducing the amount of calculation). Theoretical analysis shows that the proposed CDTZNN model (Equation 25) possesses the convergence property, and it can generate the exact solution of Equation 1) Numerical results verify the efficacy of Equation 25 and show the *O*(σ^4^) form in the SSCE of Equation 25. Simulation results under the DOBOT manipulator demonstrate the efficacy of the new motion planning scheme (Equation 35) and the applicability of the proposed CDTZNN model (Equation 25). One future research direction is to extend the proposed CDTZNN model (Equation 25) to solve the TVNE with constraints (Guo et al., 2025). Another direction is to design new CDTZNN models to solve the TVNE with noise (Liao et al., 2022; Wei and Jin, 2024). By following this work, the proposed CDTZNN model (Equation 25) will be studied by implementing on practical robot manipulators. Besides, the CDTZNN method can be further investigated and extended to underwater acoustic sensor networks (Liu J. et al., 2024), multirobot systems (Liu M. et al., 2024), inter-robot management (Liao et al., 2024), and portfolio management (Cao et al., 2025).

## Data Availability

Publicly available datasets were analyzed in this study. This data can be found here: https://cam-can.mrc-cbu.cam.ac.uk/dataset.

## Appendix

In this appendix, the derivation of the DTZNN model (Equation 24) for Jacobian matrix inversion is provided.

In Guo et al. (2017), the following CTZNN model is presented for computing the inverse of a time-varying matrix *A*(*t*) ∈ ℝ^*n*×*n*^:

(36)
$$\begin{array}{l} \dot{M}\left(t\right)=-M\left(t\right)\dot{A}\left(t\right)M\left(t\right)-\mu M\left(t\right)(A\left(t\right)M\left(t\right)-I), \end{array}$$

where *M*(*t*) ∈ ℝ^*n*×*n*^ denotes the state matrix with Ṁ(*t*) as its time derivative, Ȧ(*t*) denotes the time derivative of *A*(*t*), and μ > 0 ∈ ℝ denotes the design parameter for scaling the convergence rate of Equation 36.

Using the difference formula (Equation 21) for discretizing the CTZNN model (Equation 36) yields the expression as follows:

(37)
$$\begin{array}{l} M_{k+1}=\frac{1}{36}M_{k}+\frac{1}{2}M_{k-1}+\frac{3}{4}M_{k-2}-\frac{5}{18}M_{k-3}\\ \;-\frac{13}{6}\sigma M_{k}\dot{A}\left(t_{k}\right)M_{k}-hM_{k}(A\left(t_{k}\right)M_{k}-I)+\text{O}\left(\sigma^{4}\right), \end{array}$$

where **O**(σ^4^) ∈ ℝ^*n*×*n*^ denotes the matrix with every element being *O*(σ^4^). By eliminating **O**(σ^4^) from Equation 37, the following DTZNN model for time-varying matrix inversion is obtained:

(38)
$$\begin{array}{l} M_{k+1}=\frac{1}{36}M_{k}+\frac{1}{2}M_{k-1}+\frac{3}{4}M_{k-2}-\frac{5}{18}M_{k-3}\\ \;-\frac{13}{6}\sigma M_{k}\dot{A}\left(t_{k}\right)M_{k}-hM_{k}(A\left(t_{k}\right)M_{k}-I), \end{array}$$

which has a truncation error of **O**(σ^4^).

With regard to the Jacobian matrix inversion in this study, Ȧ(*t*_*k*_) and *A*(*t*_*k*_) in Equation 38 are replaced by $\dot{J}\left({\dot{\text{x}}}_{k},{\text{x}}_{k},t_{k}\right)$ and *J*(**x**_*k*_, *t*_*k*_), respectively. Thus, the formulation is obtained as follows:

$$\begin{array}{l} M_{k+1}=\frac{1}{36}M_{k}+\frac{1}{2}M_{k-1}+\frac{3}{4}M_{k-2}-\frac{5}{18}M_{k-3}\\ \;-\frac{13}{6}\sigma M_{k}\dot{J}\left({\dot{\text{x}}}_{k},{\text{x}}_{k},t_{k}\right)M_{k}-hM_{k}(J\left({\text{x}}_{k},t_{k}\right)M_{k}-I), \end{array}$$

which is exactly the DTZNN model (Equation 24) for Jacobian matrix inversion. On the basis of the aforementioned derivation, the truncation error of Equation 24 is **O**(σ^4^).
